# Association of β2-adrenergic receptor and insulin receptor substrate-1 polymorphisms with obesity in a Northern Indian population

**DOI:** 10.4103/0971-6866.44105

**Published:** 2008

**Authors:** Neena Srivastava, B. R. Achyut, Jai Prakash, C. G. Agarwal, D. C. Pant, Balraj Mittal

**Affiliations:** Department of Physiology, Chatrapati Shahuji Maharaj Medical University Uttar Pradesh, Lucknow, (UP) India; 2 Department of Medicine, Chatrapati Shahuji Maharaj Medical University Uttar Pradesh, Lucknow, (UP) India; 1 Department of Genetics, Sanjay Gandhi Postgraduate Institute of Medical Sciences, Lucknow, India

**Keywords:** Association, genetic polymorphism, hormones, obesity

## Abstract

**BACKGROUND:**

Imbalance in hormonal levels, regulated by host genetic factors, are known to be a major cause of obesity. Therefore, we aimed to evaluate association of genetic polymorphisms of β_2_-adrenergic receptor *(*β*_2_-AR*) and insulin receptor substrate-1 (*IRS-1*) with hormonal levels in northern Indian obese.

**METHODS:**

A total of 111 obese and 89 age matched non-obese subjects were studied after taking detailed clinical profile. Hormonal assays in serum/plasma for different hormones were done using IRMA and RIA kits. Genetic analysis of β*_2_-AR* (-47 and -20, T to C) and *IRS-1* (Arg972Gly) was done using PCR-RFLP.

**STATISTICAL ANALYSIS:**

Statistical analysis was performed by SPSS (version 11.5) software. All continuous variables were expressed as mean ± SD and tested by ANOVA test. Comparisons of categorical variables were assessed using *X*^2^ tests or Fisher's exact test. *P*-value <0.05 was considered as significant.

**RESULTS:**

Analysis showed that obese subjects had significantly higher value of blood pressure (systolic), WHR, leptin insulin and glucagon and lower value of GH. In β*_2_-AR* (-47) T/C and *IRS-1* Gly972Arg gene polymorphisms we did not found significant differences in genotype or allele frequencies. Moreover, none of the studied hormonal or metabolic parameters showed any association with the gene polymorphisms.

**CONCLUSIONS:**

Study reveals no significant association of β_2_*-AR* (-47 and -20, T to C) and IRS-1 Gly 972 Arg polymorphisms with obesity in northern Indians.

## Introduction

Obesity has become a global pandemic and long standing obesity is often among one of the risk factors for metabolic syndrome. Though studies have been performed on causation of obesity but none of the researchers showed association of such a large range of factors together. Obesity being a multifactorial disorder, in the present study we tried to associate various demographic and hormonal factors with obesity. Further, the study was extended to confirm the existence of genetic factors with obesity. In this study, we want to explore the relationship between all these demographic, hormonal, and genetic factors in obese north Indians.

Obesity is considered to be a complex trait, influenced by both environmental and host factors. Host factors include different metabolic factors and hormones that play a vital role in regulation of obesity. Female gender and older age are known to be a risk factor for obesity.[1,2] Insulin, leptin, growth hormone, thyroid hormones, and glucagon have a great influence in causation of obesity. Insulin resistance and its pathophysiologic sequalae include hypertension, dyslipidemia, atherosclerosis (metabolic syndrome or syndrome X).[3] Hyperinsulinemia contributes to the characteristic alterations in plasma lipid profile. Studies have also highlighted the importance of abdominal subcutaneous fat as an independent marker of insulin resistance.[4–6]

Leptin is a hormone released from the adipocytes which influences energy balance, exerts long acting effects to reduce adiposity by decreasing appetite and increasing thermogenesis.[2] A glucagon level regulates orexigenic-signal terminating meals after feeding.[7] In recent study, a positive association between higher fasting plasma glucagon like peptide and fat oxidation was observed thus reducing obesity.[8] Growth hormone secretion is impaired with any secretory defect of pituitary which is proportional to degree of obesity. There is conflicting information on interaction of growth hormone and its relation to cardiovascular risk and obesity.[9]

Hypertension is being observed in obese subjects with high leptin levels.[10] Recent studies have shown increased expression and activation of the circulating vasoconstrictor enzymes in adipose tissue, elevating the blood pressure in obese individuals.[11] Thyroid hormones play an important role in regulating energy homeostasis by stimulating expression of adrenergic receptor by enhancing responsiveness of catecholamines and thus regulating obesity.[12]

Apart from these factors, role of genetic polymorphisms has also been known to affect obesity phenotype. Kentaro *et al*.[13] were the first to identify two promoter polymorphisms (T to C) at -47 and -20 in 5’ leader cistron of β*_2_-AR* gene and observed the high frequency of variant allele (-47 C) in obese as compared with non-obese subjects. After this report, known to our best, no study has been published so far showing the association of these promoter polymorphisms with obesity or other conditions. *IRS-1*, principal substrate for insulin and insulin like growth factor (IGF-1) receptors is involved in glucose clearance[14,15] and is an attractive candidate gene to harbor genetic variation that might influence insulin resistance and obesity. Glycine to arginine amino acid substitution at 972 codon of its gene product leads to reduced activity.

Although, several reports on obesity has been published but none of them studied such a wide range of factors together which are involved in causation of obesity. In the present study, we aimed to find out the association of demographic, hormonal and genetic factors like β*_2_-AR* (-47 and -20 T/C) and *IRS-1* Gly972Arg gene polymorphisms with obesity in north Indian population.

## Materials and Methods

### Subjects

A total of 534 subjects were enrolled initially from the out patients department of Chatrapati Shahuji Maharaj Medical University, Lucknow and volunteers from general population of Lucknow (North India). Out of these, only 111 obese and 89 non-obese individual were selected befitting the strict inclusion criteria. All subjects were asked for detailed clinical history and required measurements were done for height, weight, body mass index (BMI) and waist-to-hip ratio. Subjects were considered as normal if they fall in normal ranges of various parameters. For example, growth hormone (GH) = 0-14 IU/ml, leptin=2-11 mg/ml, glucagon=50-150 pg/ml, serum insulin=0-30 µU/ml. Only non-smoker, non-diabetic, normotensive subjects who did not have history of coronary artery disease, neoplasia, congenital and mental disorders, and endocrine disorders like Myxoedema and Cushing syndrome were included. Study was approved from the ethical committee of the institute.

### Sample collection

After an informed consent, overnight fasting blood samples (5 ml) were taken from all subjects. Two ml blood was taken in EDTA for analysis of DNA. The genomic DNA was extracted from peripheral blood leucocytes pellet using the standard salting out method.[16] Remaining 3 ml blood was used for serum/plasma isolation.

### Hormonal assays

Assays for glucagon and leptin hormones were done in serum/plasma by radio-immunoassay using RIA kit (Linco Research, USA).[17] Insulin hormone was assayed using RIA Kit [BARC, India]. GH assay was done using hCG [^125^I] IRMA kit (RK-5CT) [IZOTOP, Budapest].[18] Blood sugar was assayed by Glucose oxidase-Peroxidase (GOD-POD) method.[19]

### Genotyping for β2-AR (-20 and -47 C/T) Polymorphism

A fragment of 353 bp in promoter of the β*_2_-AR* gene was amplified by polymerase chain reaction (PCR) using primers forward 5’- GAA TGA GGC TTC CAG GCG TC-3’ and reverse 5’- GGC CCA TGA CCA GAT CAG CA-3’.[13] Each amplification was performed using 200ng of genomic DNA in a volume of 50 µl using 25 pmol of each primer, 200 µM each dNTPs, 15 mM MgCl_2_, 100 mM Tris and 1.5 units of *Taq* polymerase (Bangalore Genei, Bangalore). DNA templates were initially denatured at 95°C for three minutes, followed by 30 cycles with denaturation at 95°C for 30 sec, annealing at 60°C for 30 sec and, extension at 72°C for 45 sec and finally, an extension at 72°C for five minutes. T to C variation at promoter sites -47 and -20 create a restriction site for *MspA1I* and *HphI* (NEB) restriction enzymes respectively. The PCR products were subjected to digestion with restriction endonucleases at 37°C for overnight. Digested products were run on 15% polyacrylamide gel.

### Genotyping for IRS-1 Gly 972 Arg polymorphism

The primers used to analyze the *IRS-1* gene were as follows: forward 5’- GCA GCC TGG CAG GAG AG-3’ and reverse 5’- CTC ACC TCC TCT GCA GC-3’.[20] The PCR products were initially denatured at 95°C for five min, cycling conditions were 95°C for one minute, 58°C for one minute, 70°C for one minute (30 cycles) followed by final extension at 72°C for 10 min. The GGG to AGG substitution at codon 972 creates a restriction site for *BstNI* (NEB) restriction enzyme. PCR product of 221 bp was digested with restriction enzyme (10 U) at 37°C for overnight and digested products were run on 15% polyacrylamide gel at 300V.

Gels were stained with ethidium bromide and visualized under ultraviolet light. All PCR reactions were performed in a Thermal Cycler (MJ Research Inc, Waltham MA). Gel documentation was done by Alphaimager™ 1220, Alpha Innotech Corporation, USA.

### Statistical analysis

Statistical analysis was performed by SPSS (version 11.5) software. All continuous variables were expressed as mean ± SD and tested by ANOVA test. Comparisons of categorical variables were assessed using χ^2^ tests or Fisher's exact test. *P*-value <0.05 was considered as significant.

## Results

The demographic and clinical profiles are shown in Table 1. A total of 200 individuals (89 non-obese and 111 obese) with BMI (25.69±5.27 kg/m^2^) waist to hip ratio (WHR) (0.87±0.08) and mean age 31.38±11.70 years were included in the present study.

**Table 1:** Demographic and clinical profile of subjects

Clinical parameters	Subjects		*P*-value
		
	Non-obese (n=89)	Obese (n=111)	
Age	36.20±9.32	35.90±12.65	NS
WHR	0.84±0.07	0.91±0.08	0.0001
Systolic BP	114.50±12.85	118.40±12.16	0.028
Diastolic BP	75.90±7.02	77.70±7.66	NS
Leptin	4.70±3.25	20.43±5.51	0.0001
Insulin	15.52±4.43	34.69±6.76	0.0001
Glucagon	71.47±29.35	93.54±34.88	0.0001
GH	1.63±0.43	0.63±0.07	0.003

All values are expressed in Mean±SD

BMI= Body mass index (kg/m^2^), WHR= Waist to hip ratio, BP= Blood pressure (mmHg), Leptin (mg/ml), Insulin (µU/ml), glucagons (pg/ml), Growth Hormone (µIU/ml), NS=Not Significant.

### Association of BMI with demographic and hormonal profile

Subjects were categorized in 2 groups according to BMI 14-24.99 (non-obese) and BMI 25-51 (obese). Further, association of BMI groups was determined with demographic and hormonal profile [Table 1]. In obese subjects, there were significant differences in waist to hip ratio (*P*≤0.001) and systolic blood pressure (*P*=0.028) than non-obese. Similarly, in hormones, leptin (*P*≤ 0.001), insulin (*P*≤0.001) and glucagon (*P*≤0.001), and GH (*P*=0.003) were associated with obesity.

### Frequency distribution of IRS-1 Gly972Arg (G/A), β_2_-AR -20 (MspA1) and β2-AR -47 (Hph1) (T/C) gene polymorphism in non-obese and obese

In Table 2, we present genotypic data for the studied polymorphisms. The polymorphism distribution was not significantly different for case and control subjects, with the TT genotype of β*_2_-AR* -20 (MspA1) gene, found in 75.68% of the obese subjects and in 79.78% of the controls, and the CT genotype found in 24.32% of the obese subjects and 20.22% in non-obese individuals.

**Table 2:** Frequency distribution of *IRS-1* Gly972Arg (G/A), β*_2_-AR* -20 (Mspa1) and β*_2_-AR* -47 (Hph1) (T/C) gene polymorphism between non-obese and obese subjects

	Non-obese (n=89)	Obese (n=111)	*P*-value
β_2_-AR -20 ((Mspa1) gene polymorphism			
TT	71 (79.78)	84 (75.68)	NS
CT	18 (20.22)	27 (24.32)	NS
β_2_-AR -47 (Hph1) gene polymorphism			
TT	63 (70.79)	84 (75.68)	NS
CT+CC#	27 (30.33)	27 (24.32)	NS
IRS-1 gene polymorphism			
GG	84 (94.38)	109 (98.20)	NS
GA	5 (5.62)	2 (1.80)	NS

* All *P*-values (2 tailed) are calculated using Fisher’s exact test or χ^2^ test (Yates corrected)

# CC count (1) was added with CT due to low sample size, NS=Not Significant, Figures in parentheses are in percentage.

The frequency of variant allele was very low. We found only one variant genotype (CC) so we include this in the heterozygous CT genotype. There was no significant difference in the frequencies of the TT genotype and CT genotype of β*_2_-AR* -47 (Hph1) gene polymorphism between the non-obese and obese subjects.

The frequency of GG, GA genotype of *IRS-1* Gly972Arg gene polymorphism was also not significantly different in non-obese and obese subjects. Here also, no variant AA was found in non-obese and obese subjects.

### Association of IRS-1 Gly972Arg and β_2_-AR -20 and -47 T/C polymorphisms with demographic and hormonal profile

In β*_2_-AR* gene polymorphism (-47, -20), blood pressure (systolic and diastolic), WHR (waist to hip ratio) leptin, insulin, glucagon, GH, T3, T4, and TSH levels were not significantly different in non-obese and obese subjects (*P*>0.05) [Tables 3 and 4]. We did not find any significantly difference in *IRS-1* gene polymorphism (Gly/Arg genotype) for demographic and hormonal profile in non-obese and obese subjects. There was no influence of *IRS-1* gene polymorphism on demographic (*P*>0.05) and hormonal profile (*P*>0.05) [Table 5].

**Table 3:** Association of β_2_*-AR* (-20 T>C) polymorphism with clinical parameters

Variables	Non-obese β_2_ Adrenergic receptor		*P*-value	Obese β_2_ Adrenergic receptor		*P*-value
				
	-20TT (n=71)	-20CT+CC (n=18)		-20TT (n=84)	-20CT+CC (n=27)	
WHR	0.84±0.09	0.84±0.07	NS	0.91±0.08	0.93±0.01	NS
Systolic BP	118.22±9.52	122.86±4.30	NS	128.10±9.60	128.20±11.36	NS
Diastolic BP	79.33±7.71	83.14±6.62	NS	87.33±9.59	88.40±8.96	NS
**Hormonal profile**						
Leptin	13.96±4.56	14.77±3.88	NS	17.97±5.29	16.18±4.03	NS
Insulin	21.20±6.49	17.43±6.21	NS	27.36±5.85	25.53±4.21	NS
Glucagon	86.78±31.73	80.86±22.43	NS	107.03±32.84	113.45±34.02	NS
GH	1.82±0.07	1.39±0.06	NS	0.61±0.05	0.53±0.04	NS

WHR= Waist to hip ratio, BP= Blood pressure (mmHg),Leptin (mg/ml), Insulin(µU/ml), glucagons(pg/ml), Growth Hormone(ng/ml), NS=Not Significant.

**Table 4:** Association of β_2_-AR (-47 T>C) polymorphism with clinical parameters

Variables	Non-obese β_2_ Adrenergic receptor		*P*-value	Obese β_2_ Adrenergic receptor		*P*-value
				
	-47TT (n=63)	-47CT+CC (n=26)		-47TT (n=84)	-47CT+CC (n=27)	
WHR	0.85±0.09	0.81±0.08	NS	0.91±0.08	0.93±0.08	NS
Systolic BP	119.04±7.98	119.56±11.48	NS	127.39±8.98	130.34±12.54	NS
Diastolic BP	79.92±7.25	80.66±8.83	NS	86.70±8.62	90.29±11.22	NS
**Hormonal profile**						
Leptin	15.11±4.84	17.50±4.99	NS	17.20±4.48	18.55±3.52	NS
Insulin	19.98±5.21	21.67±4.03	NS	26.60±4.62	27.89±7.93	NS
Glucagon	86.68±28.66	91.89±34.98	NS	110.82±32.83	101.76±33.65	NS
GH	1.70±0.06	1.82±0.07	NS	0.61±0.05	0.53±0.05	NS

WHR= Waist to hip ratio, BP= Blood pressure (mmHg), Leptin (mg/ml), Insulin (µU/ml), glucagons (pg/ml), Growth Hormone (ng/ml), NS=Not Significant.

**Table 5:** Association of IRS-1 (Gly972Arg) polymorphism with obesity

Variables	Non-obese IRS-1		*P*-value	Obese IRS-1		*P*-value
		
	Gly/Gly (n=84)	Gly/Arg (n=5)		Gly/Gly (n=109)	Gly/Arg (n=2)	
WHR	0.87±0.08	0.85±0.07	NS	0.88±0.09	0.89±0.04	NS
Systolic BP	77.1±9.03	80.0±14.14	NS	82.48±11.20	80.00±0.00	NS
Diastolic BP	116.05±10.95	122.00±12.75	NS	123.20±13.15	120.00±0.00	NS
**Hormonal profile**					
Leptin	11.647±2.23	8.93±2.22	NS	14.93±2.25	20.00±0.00	NS
Insulin	21.83±7.95	17.75±8.34	NS	26.60±9.77	25.50±7.67	NS
Glucagon	73.52±22.34	72.75±21.57	NS	84.30±19.15	100.00±2.42	NS
GH	0.93±0.05	1.06±0.09	NS	0.62±0.06	0.52±0.02	NS

WHR= Waist to hip ratio, BP= Blood pressure (mmHg), Leptin (mg/ml), Insulin (µU/ml), glucagons (pg/ml), Growth Hormone (ng/ml), NS=Not Significant.

## Discussion

The combined effects of genes, environment, and lifestyle are responsible for development of obesity. In this study, BMI was taken as major criteria of obesity and correlation of BMI with different clinical and genetic parameter was seen like waist to hip ratio and hormonal levels (insulin, leptin, glucagon, and growth hormone).

A statistically significant (*P*≤0.001) association of increased leptin levels (20.4±5.5 vs. 4.7±3.3) was observed in subjects with high BMI. The leptin level was significantly higher in obese females. These results are similar to the findings of Considine *et al*.[1] where leptin levels were 31.3±24.1 ng/ml in obese subjects and 7.5± 9.3 ng/ml in normal weight subjects (*P*<0.001). Richard *et al*.[21] also found increased leptin levels in obese group (17.1± 4.8 vs. 5.8± 1.42 ng/ml, *P*=<0.001) than normal subjects. Recent studies also revealed a correlation of BMI with increased leptin in obese women.[2] Subjects with higher BMI and WHR showed higher leptin levels in present study. This was in agreement (*P*<0.001) with the finding of previous study conducted by Paul *et al*.[22] Richard *et al.*[21] reported negative correlation between waist to hip ratio and leptin levels, which was not statistically significant (*P*=0.99).

GH level showed a significant fall with obesity in our study and these findings are in conformity with the observations of a recent study.[9] Savastano *et al*.[23] observed a negative correlation with age, BMI, waist circumference and fat mass which also favors this study.

In this study, we investigated 2 genetic variants of the β*_2_ AR* gene and 1 genetic variant of *IRS-1* gene as candidates to predispose obesity. Blood pressure as well as fat metabolism are regulated by the β*_2_ AR*, so we tested the β*_2_ AR* polymorphisms for association with hypertension obesity. Genetic variance in the *IRS-1* is thought to play a key role in the insulin resistance that characterizes type 2 diabetes.[24]

So, we wanted to look for association of the polymorphisms in *IRS-1* and β*_2_-AR* genes. Our results showed that Gly to Arg variation in *IRS-1* gene was observed in 7 (3.5%) subjects. Clausen *et al*.[25] found the increased insulin resistance with *IRS-1* Gly 972 Arg polymorphism in heterozygous state in obese patients. Caucasian study performed in two cohorts found the increased insulin resistance in obese children.[26] Siqal *et al*.[27] reported that Gly to Arg polymorphism predisposes to NIDDM only in the presence of excess of body weight.

Insulin promotes adipocyte triglyceride stores by a number of mechanisms stimulating tri-glyceride synthesis (lipogenesis). In adipocytes of obese human *IRS-1* protein expression is down regulated, resulting in decreased *IRS-1* associated phosphoinisitide 3 kinase (PI3K) activity which comes down stream in the insulin metabolism pathway and has antilipolytic action which is preserved in diabetic obese despite of low insulin levels resulting in maintenance or expansion of fat stores.

Studies have shown that β_2_-adrenergic receptor (ADRB2) controls energy balance and storage of fat. This receptor is down regulated in white adipose tissue in obesity. Its gene is known to be highly polymorphic.[28] Several polymorphisms in coding region and their haplotypes have been found to be associated with asthma, hypertension, diabetes and obesity.[29–32] The most common polymorphism Arg16Gly was found to be associated with risk for obesity.[33] A study from western world showed that Arg16Gly polymorphism was associated with weight gain from childhood to young adulthood in males.[34] However, some contradictory observations have been seen in some of the studies.[35] Earlier study published from Japan[13] stated the importance of promoter polymorphisms in β*_2_-AR* gene. Therefore, to find out if any association is present in Indian population, we performed the present study. However, this study also did not reveal any significant association of β*_2_-AR* -20 and -47 T/C polymorphisms with obesity.

In conclusion, the present study revealed significant association of demographic and hormonal factors with obesity in north Indians. No significant association of any obesity related factor could be established with β*_2_-AR* promotor polymorphisms and *IRS-1* Gly 972 Arg polymorphism. The limitations of this study is low sample size and the subjects consider as obese have BMI ≤25 rather then BMI ≤30. Moreover, frequencies of variant alleles in these polymorphisms were very low, and large sample size may be required to achieve definitive results of the association.
